# Synergistic effect of ginsenoside Rg3 with verapamil on the modulation of multidrug resistance in human acute myeloid leukemia cells

**DOI:** 10.3892/ol.2014.1826

**Published:** 2014-01-24

**Authors:** SUNG SU KIM, SIN SEONG, SUNG YOUNG KIM

**Affiliations:** 1 Department of Oriental Medicine, Kyung Hee University College of Oriental Medicine, Seoul 130-701, Republic of Korea; 2 Department of Biochemistry, School of Medicine, Konkuk University, Seoul 143-701, Republic of Korea

**Keywords:** ginsenoside, Rg3, verapamil, multidrug resistance, acute myeloid leukemia

## Abstract

The pharmacological modulatory effects of 20(S)-ginsenoside Rg3 (20S-Rg3) on multidrug resistant cancer cells are reported in the present study. The effects of 20(S)-Rg3 on the modulation of doxorubicin (DOX) and vincristine (VCR) resistance were examined in the HL60 multidrug resistant subline of human acute myeloid leukemia cells. Results demonstrated that 20S-Rg3 is as effective as verapamil (Vp) for modulating the high degree primary DOX resistance and low degree VCR cross-resistance expressed by the H160 cell line. Furthermore, the present study demonstrates for the first time, using isobologram analysis, that the combination of 20S-Rg3 and Vp enhances the reversal of DOX and VCR resistance in a supra-additive or at least an additive manner. These results indicate that 20S-Rg3 may be used as a Vp synergizer or as a promising alternative to Vp in the chemosensitization of multidrug resistant acute myeloid leukemia, with far fewer side effects.

## Introduction

Multidrug resistance (MDR) is a major obstacle to the effectiveness of currently available cytotoxic drugs. Although various mechanisms of MDR have been reported, the exact mechanisms of resistance remain unclear. The pharmacological mechanism of MDR appears to be a reduced accumulation of intracellular drugs. This is due to enhanced drug efflux by energy-dependent transmembrane transporters, including P-glycoprotein (P-gp), MDR protein-1 and other similar pumps. In the past few decades, a large number of multidrug efflux pump inhibitors have been described and tested (reviewed in 1 and 2). However, the dose of the efflux pump modulator used in clinical studies has been limited by significant side effects, for example cardiac toxicity.

**Figure 1:**
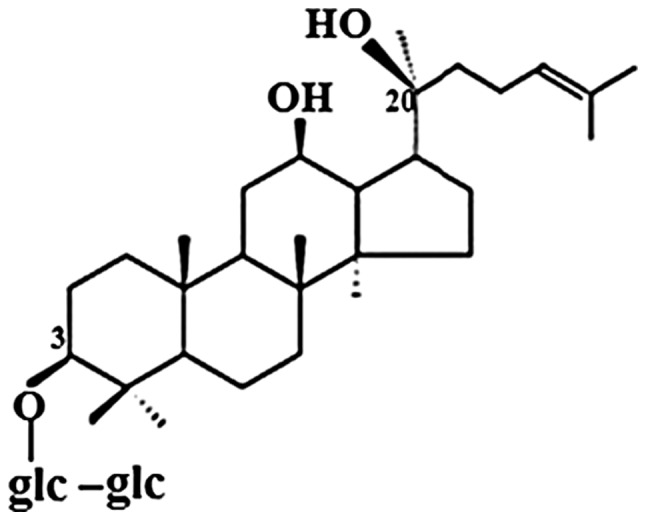
Chemical structure of 20(S)-Rg3. 20(S)-Rg3, 20(S)-ginsenoside Rg3; glc, β-*o*-glucopyranosyl.

20(S)-ginsenoside Rg3 [20(S)-Rg3] is a chemical component extracted from *Panax ginseng* and has a molecular weight of 784.3 Da (3). Ginsenoside has a four-ring steroid-like structure (Fig. 1) and exhibits a number of biological activities. Previously, 20(S)-Rg3 has been shown to modulate the MDR phenotype (4–7). However, the effects of a combination of 20(S)-Rg3 and other modulators on the MDR phenotype have not been studied. The present study used doxorubicin (DOX) and vincristine (VCR) resistance in human MDR cell lines to evaluate the combined use of 20(S)-Rg3 and verapamil (Vp), a well-known MDR inhibitor. It was aimed to determine whether an interaction between the two compounds would allow lower concentrations to be used clinically to modulate drug resistance.

## Materials and methods

### Cell lines and materials

Human acute myeloid leukemia HL60 cell lines were purchased from the American Type Culture Collection (Rockville, MD, USA). The DOX-resistant cell line (HL60/DOX) was developed by stepwise exposure of HL60 to increasing drug concentrations, as previously described (8). The resistant variants demonstrated increased expression of P-gp. Cells were grown in RPMI 1640 medium (Gibco-BRL, Carlsbad, CA, USA) supplemented with 10% fetal calf serum, 100 U/ml penicillin and 5 μg/ml gentamicin at 37°C in 5% CO_2_. Prior to use, the resistant cells were grown in drug-free medium over several generations. DOX, VCR, 20(S)-ginsenoside Rg3 and Vp were obtained commercially from Sigma-Aldrich (St. Louis, MO, USA).

### Measurement of growth by XTT cell proliferation assay

Dose response curves for DOX-resistant cell lines were determined by XTT cell proliferation assay (Roche Diagnostics, Inc., Mannheim, Germany). The cell proliferation was based on the ability of the mitochondrial succinate-terazolium reductase system to convert yellow tetrazolium salt XTT [sodium 3′-(1-[phenylaminocarbonyl]-3,4-tetrazolium)-bis (4-methoxy-6-nitro) benzene sulfonic acid hydrate] to orange formazan dye. In short, cells (10^4^ cells/well) were plated into 96-well plates containing 200 μl growth medium in the absence or presence of increasing concentrations of the anticancer agents at 37°C and 5% CO_2_. This was subsequent to verification of cell viability by trypan blue dye exclusion assay. Following an incubation period of 48 h, freshly prepared XTT reagent was added to each well as specified by the manufacturer’s instructions. The optical density was measured at 490 nm in a VersaMax ELISA plate reader (Molecular Devices, Sunnyvale, CA, USA). The reference wavelength used was 650 nm. Subsequently, the 50% inhibitory concentrations (IC_50_) of the compounds were calculated from cell proliferation plots (Table I). The HL60 isolates were 83-fold more resistant to DOX than the sensitive parent cell lines. These cells also exhibited 9.8-fold more cross-resistance to VCR than the sensitive parent cell lines (Table I). In the present study, an incubation dose of 1 μM DOX or 10 nM VCR was used for comparison with 20(S)-Rg3 and/or Vp-induced reversal of multidrug resistance.

### Isobologram

**Figure 2:**
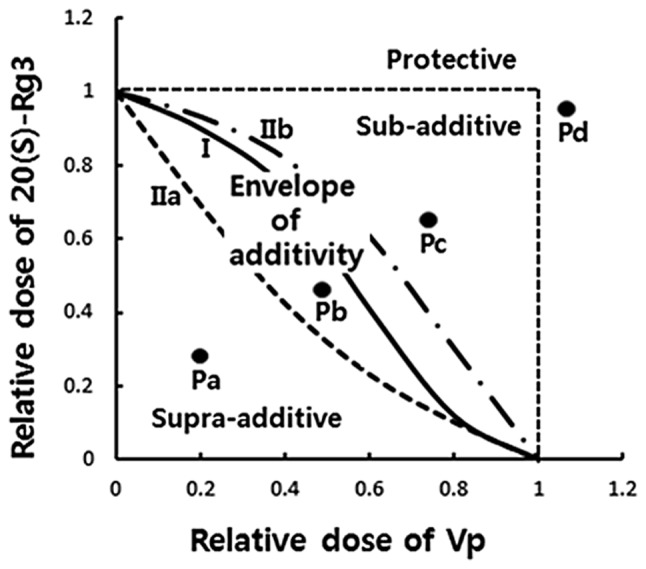
Schematic representation of isobologram analysis. Envelope of additivity is surrounded by mode I (solid line) and II (dotted lines) isobologram lines, constructed from the dose-response curves of 20(S)-Rg3 and Vp. The concentrations that produced 50% cell growth inhibition were expressed as 1.0 on the x- and y-axis of the isobolograms. The experimental data points, Pa, Pb, Pc, and Pd, represent the supraadditive, additive, subadditive and protective effects, respectively. 20(S)-Rg3, 20(S) ginsenoside Rg3; Vp, verapamil.

For drug interaction analysis, the isobologram method of Steel and Peckham was used (9). The theoretical basis of the isobologram method has been described in detail previously (10,11). This method has the advantage of being independent of the mechanism of action and the shape of the dose-response curve (9,12). The isobologram shows the relationship of the dose of 20(S)-Rg3 and Vp in achieving a 50% reversal of drug resistance. Using previously described methods (9), isoeffect lines were constructed for the combined effects of the two drugs. If 20(S)-Rg3 and Vp were found to act additively by independent mechanisms, the doses that together produced 50% resistance were plotted as mode I. When the dose of the first modulator (mode IIa, 20(S)-Rg3; mode IIb, Vp) had been chosen, the isoeffect line was calculated by taking the dose increment of the second modulator (mode IIa, Vp; mode IIb, 20(S)-Rg3) that gave the required contribution to the total effect up to the limit. When the experimental isoeffect points fell within the area surrounded by three lines (envelope of additivity), the combination was regarded as additive. When the data points fell to the left or the right of the envelope, the drugs were regarded as having a supra-additive (synergistic) or sub-additive effect (antagonistic), respectively (Fig. 2).

### Measurement of growth by 5-bromo-2′-deoxyuridine (BrdU) cell proliferation assay

The BrdU incorporation assay was performed using the BrdU proliferation assay kit, according to the manufacturer’s instructions (Calbiochem, La Jolla, CA, USA). Briefly, cells were seeded onto 96-well plates in RPMI 1640 medium containing 10% fetal bovine serum (FBS). Medium was replaced 24 h later with RPMI 1640 medium containing 2% FBS, in the presence or absence of a drug. Plates were labelled for 2 h with BrdU, followed by fixation for 30 min and incubation with primary anti-BrdU monoclonal and secondary peroxidase-conjugated goat anti-mouse antibodies, then developed using colorimetry. Absorbance at 460 nm was measured using the VersaMax ELISA plate reader.

## Results

**Figure 3:**
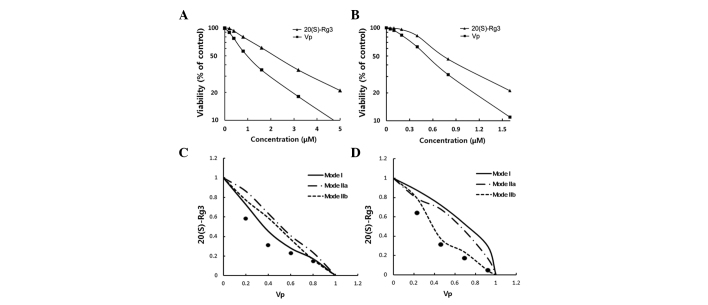
Synergistic effects of 20(S)-Rg3 and Vp on the reversal of DOX and VCR resistance. (A and B) Dose-response curves for the effect of 20(S)-Rg3 and Vp on the reversal of DOX/VCR in HL60/DOX cells. (C and D) Dose-normalized 50% isobologram analysis for HL60/DOX cells treated with 1 μM DOX or 8 nM VCR. The interaction of 20(S)-Rg3 and Vp was analyzed by an ‘envelope of additivity’, consisting of lines for modes I (solid line), IIa (dot-dashed line) and IIb (dashed line). Block dots represent observed data. 20(S)-Rg3, 20(S) ginsenoside Rg3; Vp, verapamil; DOX, doxorubicin; VCR, vincristine.

**Figure 4:**
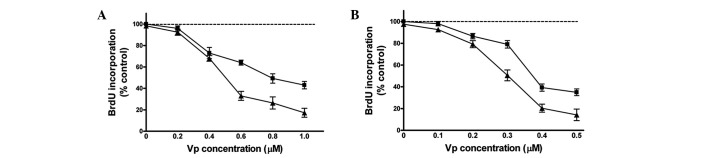
Enhancement of Vp activity by 20(S)-Rg3. (A) HL60/DOX cells were treated with a 0.3 μM fixed concentration of 20(S)-Rg3 (dotted line), increasing concentrations of Vp (■), or a combination of Vp plus 0.3 μM 20(S)-Rg3 in the presence of 1 μM of DOX (▲). (B) HL60/DOX cells treated with a 0.1 μM fixed concentration of 20(S)-Rg3 (dotted line), increasing concentrations of Vp (■), or a combination of Vp and 0.2 μM 20(S)-Rg3 in the presence of 8 nM VCR (▲). BrdU incorporation was measured by ELISA. Data are presented as the mean ± standard deviation of three independent experiments. BrdU, 5-bromo-2′-deoxyuridine; Vp, verapamil; 20(S)-Rg3, 20(S) ginsenoside Rg3; DOX, doxorubicin.

Fig. 3A shows the dose-response effect of 20(S)-Rg3 and Vp on the growth-inhibitory effect of 1 μM DOX on the HL60/DOX cell line. The modulator concentration required to reach the IC_50_ value for HL60/DOX in the presence of 1 μM DOX was 0.96 and 2.2 μM for Vp and 20(S)-Rg3, respectively. Using standard methods for determining drug synergism and antagonism, an isobologram plot was constructed for 20(S)-Rg3 and Vp (Fig. 3C). The experimental isoeffect points fell along the boundaries of the additive area at lower doses of 20(S)-Rg3. At higher concentrations of 20(S)-Rg3, the isoeffect points fell to the left of the envelope of additivity, indicating a supra-additive or synergistic effect for the two modulators. The dose-response curves, constructed for the effects of varying concentrations of 20(S)-Rg3 and Vp on the reversal of VCR cross-resistance in HL60/DOX cells, are presented in Fig. 3B. The modulator concentration required to reach the IC_50_ value for HL60/DOX, in the presence of 10 nM VCR, was 0.52 and 0.73 μM for Vp and 20(S)-Rg3, respectively. The effects of combined doses of 20(S)-Rg3 and Vp on the reversal of VCR resistance are shown in Fig. 3D. The experimental isoeffect points fall to the far left of the area of additivity, indicating a clear supra-additive effect of 20(S)-Rg3 and Vp in the reversal of VCR resistance. The synergistic interaction of 20(S)-Rg3 and Vp was further examined by measuring the effect of Vp on BrdU incorporation at a fixed concentration of 20(S)-Rg3. As shown in Fig. 4A and B, a low concentration of 20(S)-Rg3 had little effect on BrdU incorporation in the HL60/DOX cell line in the presence of a fixed concentration of DOX (1 μM) or VCR (10 nM). However, as the concentration of Vp increased, a clear synergistic interaction was observed between the two agents.

## Discussion

In general, agents used to reverse MDR are termed ‘MDR modulators’ or ‘chemosensitizers’. *In vitro*, a number of agents, including calcium channel blockers, calmodulin antagonists, cyclosporines, noncytotoxic anthracycline, vinca alkaloid analogs, steroids, dipyridamole and miscellaneous cationic compounds, have been demonstrated to modulate the expression of the MDR phenotype (13). Vp, a phenylalkylamine calcium channel blocker, is the first reported chemosensitizer that inhibits MDR (14) and the effects of this agent have been demonstrated in a previous clinical study (15). Vp and its analogues partially reverse the resistance of numerous MDR cell lines by decreasing drug efflux and increasing intracellular drug accumulation (13). However, the relatively broad spectrum of toxicity of Vp and its analogues has led to the development of combination therapies of Vp with other modulators.

The efficacy of combinations of chemosensitizers in reversing the MDR phenotype has been extensively studied over the past two decades. Vp and cyclosporin A have demonstrated a synergistic chemosensitizing interaction in human leukemia cell lines (16), whilst Vp and the thioxanthene, *trans*-flupenthixol, have shown an additive effect in an MDR breast cancer cell line (17). However, these compounds were found toxic at the doses required to attenuate drug efflux pumps (18). Therefore, improved approaches are needed to prevent MDR. A number of the emerging candidates are herbal constituents shown to inhibit the multidrug efflux pump *in vitro,* in concentrations that are compatible with clinical applicability.

Ginseng is one of the most commonly used herbal medicines and is reported to have a wide range of pharmacological and therapeutic applications (19). It has previously been demonstrated that ginsenosides, the major pharmacologically active ingredients of ginseng, possess MDR-modulating activity (20). Ginsenoside potentiates the effects of anticancer agents in multidrug resistant cells (4,7,20,21). Although the mechanisms have yet to be fully defined, it appears that ginsenoside may modulate efflux-mediated drug accumulation defects. A previous study revealed that protopanaxadiol-containing ginsenosides (Rg3 and Rh2) and protopanaxatriol-containing ginsenosides (Rg1 and Rh1) are able to inhibit breast cancer resistance proteins, a newly identified ATP-binding cassette family of drug transporters (7). It was also revealed that the majority of ginsenosides, including Rg2, Rg3, Rh1, Rh2 and Rh3, inhibited P-gp-MDR activity. Among these, 20(S)-Rg3 was found to have the most potent inhibitory activity on MDR (20). Notably, Rg3 was also shown to modulate the fluidity of the plasma membrane (6). In a study on multidrug resistant mouse lymphoma cells, Molnar *et al* demonstrated that ginsenosides, excluding Rb1, had a inhibitory effect on the drug efflux pump and increased drug accumulation in MDR cells (5). In a separate study, Kim *et al* used photo-affinity labeling of P-gp with [3H]azidopine to demonstrate that Rg3 competes with [3H]azidopine for binding P-gp (4).

The ideal reversing agents used in combination must meet two requirements. Firstly, they must lack dose-limiting toxicities and demonstrate no pharmacokinetic interactions with other drugs. Secondly, the agents must undergo additive or synergistic chemosensitizing interactions with the resistant tumor cells. As 20(S)-Rg3 appears to meet the first requirement, the effectiveness of combined chemosensitization with Vp and 20(S)-Rg3 was evaluated in an MDR human leukemia cell line model. In the present study, using the isobologram technique, the effect of the combined modulators on HL60/DOX cell high-degree DOX resistance and low-degree VCR cross-resistance was demonstrated to be supra-additive. In addition to inducing MDR reversal, ginsenosides, particularly Rg3, possess a variety of antimutagenic and cancer-inhibitory properties (22–25). This supports a new treatment paradigm which uses combinations of chemotherapy with 20(S)-Rg3 to treat MDR cancer cells.

In summary, results of the present study demonstrate that the ginseng component, ginsenoside Rg3, significantly enhances the effect of Vp on reversal of MDR of acute myeloid leukemia cells. As clinical studies of chemotherapy modulation are developed, the use of a combination of different modulators at sub-toxic doses may become of interest. Given that ginseng components are generally considered safe medicinally, their synergistic activity in reversing MDR may provide a rationale for the combined use of these agents in *in vivo* trials.

## Figures and Tables

**Table I:** Comparison of IC_50_ values of DOX/VCR in HL60/DOX and HL60 cells.

	IC_50_ (nM)		
		
Drug	HL60/DOX	HL60	Degree of resistance or cross-resistance
DOX	1411.0±342^a^	17.0±4.0	83.0
VCR	11.8±0.4	1.2±0.2	9.8

a Mean ± standard deviation of at least 3 separate experiments.

DOX, doxorubicin; VCR, vincristine.

## Abbreviations

20(S)-Rg3: 20(S)-ginsenoside Rg3
Vp: verapamil
DOX: doxorubicin
VCR: vincristine
MDR: multidrug resistance
P-gp: P-glycoprotein
BrdU: 5-bromo-2′-deoxyuridine
